# Increased levels of serum adenosine deaminase and increased risk of diabetic peripheral neuropathy in type 2 diabetes

**DOI:** 10.3389/fendo.2022.997672

**Published:** 2022-10-04

**Authors:** Chao Yu, Lei Zhuang, Feng Xu, Li-hua Zhao, Xiao-hua Wang, Chun-hua Wang, Li-yan Ning, Xiu-lin Zhang, Dong-mei Zhang, Xue-qin Wang, Jian-bin Su

**Affiliations:** ^1^ Department of Clinical Laboratory, Affiliated Hospital 2 of Nantong University, First People’s Hospital of Nantong City, Nantong, China; ^2^ Department of Endocrinology, Second People’s Hospital of Nantong City, Nantong, China; ^3^ Department of Endocrinology, Affiliated Hospital 2 of Nantong University, First People’s Hospital of Nantong City, Nantong, China; ^4^ Department of Administration, Affiliated Hospital 2 of Nantong University, First People’s Hospital of Nantong City, Nantong, China; ^5^ Medical Research Center, Affiliated Hospital 2 of Nantong University, First People’s Hospital of Nantong City, Nantong, China

**Keywords:** adenosine deaminase, neuropathy, risk factor, diagnosis, type 2 diabetes

## Abstract

**Background:**

Increased serum adenosine deaminase (ADA) levels have been shown to be involved in metabolic abnormalities and immune disequilibrium, which may in turn contribute to inflammatory diseases. This study aimed to determine whether increased serum ADA levels are related to diabetic peripheral neuropathy (DPN) in patients with type 2 diabetes (T2D).

**Methods:**

This study was part of a series exploring the potential risks for DPN. All patients received DPN assessment based on neuropathic symptoms, neuropathic signs, and nerve conduction studies to calculate the composite *Z* score of nerve latency, amplitude and conduction velocity (NCV). DPN was confirmed by both at least a presentation of neuropathic symptoms/signs and an abnormal nerve conduction index. Serum ADA levels were also synchronously detected.

**Results:**

A total of 384 eligible patients with T2D were recruited for this study, and 24.5% (n=94) were determined to have DPN. Increases in serum ADA levels were closely associated with increases in composite *Z* score of latency (*β*=0.263, *t*=5.273, *p*<0.001) and decreases in composite *Z* score of amplitude (*β*=–0.126, *t*=–2.352, *p*=0.019) and NCV (*β*=–0.201, *t*=–3.841, *p*<0.001) after adjusting for other clinical covariates. Moreover, each 5 U/L increase in serum ADA levels was associated with a 1.781-fold increased adjusted odds ratio of having DPN (95% confidence interval: 1.271–2.495). Furthermore, the optimal cut-off value of serum ADA levels to discriminate DPN was ≥14.2 U/L (sensitivity=59.57%, specificity=75.52% and Youden index=0.351) after analysis by receiver operating characteristic curve.

**Conclusions:**

Increased serum ADA levels may be a potential risk factor for DPN in patients with T2D.

## Introduction

Diabetic peripheral neuropathy (DPN), a distressing and poorly tolerated complication of diabetes, has been linked to considerable health costs and social burdens (1). DPN is the main facilitator for fall easily and subsequent fracture and diabetic foot and subsequent amputation (2). Rather than a purely sensorimotor peripheral neuropathy, DPN also involves structural alterations and functional abnormalities in the central nervous system (3–5). Moreover, DPN is not only always comorbid with cardiovascular complications and other microvascular complications in patients with type 2 diabetes (T2D) (6–8) but could also predict cardiovascular disease and all-cause mortality in these patients (9, 10). Although the pathogenesis of DPN is not fully understood, it involves the interaction between multiple risk factors. Therefore, an exploration of potential risk factors for DPN in T2D is worthwhile and may help develop approaches to prevent or ameliorate DPN.

Adenosine deaminase (ADA) is a critical enzyme for purine catabolism that is ubiquitously expressed in various tissues and catalyzes the irreversible deamination of adenosine and 2′-deoxy-adenosine to yield inosine and 2′-deoxy-inosine, respectively (11). In addition to its enzymatic property, ADA is also a costimulatory molecule that is engaged in a variety of cellular processes, such as regulation of DNA synthesis, mediation of adenosine receptor signaling and modulation of intercellular interactions (12). Increased serum ADA activity has been shown to be involved in various metabolic and inflammatory diseases, including atherosclerosis (13), thrombosis (14), acute myocardial infarction (15), hypertension (16, 17), Graves’ disease (18), and T2D (19). Metabolic abnormalities and inflammatory disorders are essential conditions for DPN in T2D (20). In light of the above, we hypothesized that increased serum ADA levels may take part in the pathogenesis of DPN in T2D.

Therefore, we designed the present study to elucidate whether increased serum ADA levels are connected to DPN in patients with T2D.

## Methods

### Participant recruitment

This study was part of a series we designed to explore the potential risks for DPN in T2D. We posted a notice to recruit participants for the study at the First People’s Hospital of Nantong City and Second People’s Hospital of Nantong City between November 2017 and December 2021. The inclusion criteria for participants were as follows (1): were between 20 and 80 years of age (2); met the diagnostic criteria for T2D (2015 Edition, American Diabetes Association) (21); and (3) voluntarily agreed to take part in the study. The exclusion criteria for participants were (1) positivity for autoantibodies associated with type 1 diabetes (2); a history of COVID-19 (3); personal history of malignancy (4); thyroid hormonal abnormality (e.g., hyperthyroidism and hypothyroidism) (5); severe cardio-cerebral vascular diseases (e.g., myocardial infarction and stroke) (6); history of vascular interventional surgery (7); peripheral artery disease (8); thrombotic diseases (9); severe chronic kidney disease, and estimated glomerular filtration rate (eGFR)<60 mL/min/1.73m^2^ (10); autoimmune diseases (11); acute or chronic infectious diseases (12); connective tissue diseases (13); use of drugs with side-effects of neurotoxicity (14); deficiencies of folate or vitamin B12 (15); neurodegenerative diseases (16); inflammatory demyelinating neuropathies; and (17) spinal or foraminal stenosis.

We ultimately recruited 384 eligible patients with T2D for this study. The study was initiated and academically supported by the First People’s Hospital of Nantong, so the study protocol was reviewed and approved by the First People’s Hospital of Nantong. In addition, the study protocol followed the Declaration of Helsinki, and all participants provided informed consent when recruited into the study.

### Clinical data collection

Clinical data from all participants were collected by trained clinical staff. These data included age, sex, body mass index (BMI), systolic/diastolic blood pressure (SBP/DBP), diabetes duration, hypertension status, current smoking status, statin use, and antidiabetic treatments. Hypertension was identified as reported in our previous studies (22, 23). Antidiabetic treatments in our study were divided into nine categories: drug naive, insulin, secretagogues, metformin, thiozolindiones (TZDs), α-glucosidase inhibitors (AGIs), dipeptidyl peptidase-4 inhibitors (DPP-4Is), sodium-glucose cotransporter-2 inhibitors (SGLT-2Is), and glucagon-like peptide-1 receptor agonists (GLP-1RAs).

Serum was isolated from fasting blood specimens (stored in CAT Serum Clot Activator tubes, Greiner Bio-one) for the measurement of ADA, alanine aminotransferase (ALT), aspartate aminotransferase (AST), gamma-glutamyl transpeptidase (GGT), total bilirubin (TBI), albumin, triglycerides (TG), total cholesterol (TC), high-density lipoprotein cholesterol (HDLC), low-density lipoprotein cholesterol (LDLC), glucose, C-peptide, uric acid (UA) and cystatin C (CysC) levels. Serum ADA was measured by the peroxidase method (Adenosine deaminase Reagent Kit, MeiKang Biotechnology Co., Ltd, Ningbo, China) in a fully automated biochemical analyser (Model 7600, Hitachi, Japan). Whole blood specimens were drawn to detect glycosylated hemoglobin (HbA1c). Plasma was also isolated to detect glucagon. Insulin sensitivity (IS) was calculated based on fasting C-peptide (CP), IS-CP=20/(fasting C-peptide[ng/ml]×fasting glucose[mmol/L]). Serum creatinine was also detected to calculate the estimated glomerular filtration rate (eGFR) using the Modification of Diet in Renal Disease equation (24).

### Screening for DPN and nerve conduction studies

Screening for DPN was carried out as reported in our previous studies (22, 25). Confirmation of DPN is dependent on both at least a presentation of neuropathic manifestations (neuropathic symptoms or signs) and an abnormal index of peripheral nerve conduction, which is based on the Toronto Consensus Guideline (26).

Neuropathic symptoms and signs were collected by detailed history taking and physical examinations. Neuropathic symptoms manifested mainly in the lower limbs, which included numbness, imbalance, paraesthesia (e.g., cooling or heat sensation, allodynia and hyperalgesia) and paroxysmal/persistent pain, such as tingling, burning, electric shock, cutting, and stabbing pain. Neuropathic signs caused by the involvement of large fibre neuropathy were assessed based on reduced or absent ankle reflexes (using an appropriate reflex hammer), vibration perception (using a 128-Hz tuning fork on the great toes), touch perception (using a 10-g monofilament on four sites per foot), balance perception and proprioception (sense of limb position). Meanwhile, neuropathic signs caused by the involvement of small fibre neuropathy were assessed based on reduced or absent thermal sensation (using cold and warm objects) and pinprick sensation (using a pin).

Nerve conduction studies were performed by an experienced neuropathic technician using an electromyogram (MEB-9200K, Nihon Kohden), including the median nerve (MN) and ulnar nerve (UN) of the bilateral upper limbs and the common peroneal nerve (CPN), posterior tibial nerve (PTN), sural nerve (SN) and superficial peroneal nerve (SPN) of the bilateral lower limbs. The nerve conduction parameters included the nerve action potential onset latency, amplitude and conduction velocity (NCV) of the MN, UN, CPN and PTN motor nerves and of the MN, UN, SN and SPN sensory nerves. Data for nerve latency, amplitude and NCV were then *Z* score transformed. Furthermore, a composite *Z* score of latency was generated by taking the average value of the latency *Z* score of all motor and sensory nerves of the upper and lower limbs, as also described in our previous study (25). In the same way, composite *Z* scores of amplitudes and NCV were generated. An abnormality of nerve conduction studies was defined by the presence of at least one abnormal nerve attribute (of latency, amplitude or nerve conduction velocity) in two or more nerves of the lower limbs and upper limbs (27).

### Statistical analysis

First, we plotted the frequency distribution of serum ADA levels and examined the characteristics of the data. The range of serum ADA levels in all recruited patients was 4.0–37.0 U/L, and those of the first, second, third and fourth quartiles (Q1, Q2, Q3 and Q4) were 4.0–9.5 U/L, 9.6–12.1 U/L, 12.2–15.9 U/L and 16.0–37.0 U/L, respectively. Descriptive statistics were performed for all patients and four subgroups categorized by the quartiles of serum ADA levels. Mean and standard deviation are used for normally distributed quantitative data, median and interquartile range for skew-distributed quantitative data, and frequency and percentage for qualitative data. To analyse changes in trends of clinical data among the four subgroups, one-way analysis of variance (ANOVA) with linear polynomial contrasts (*F* value), the Jonckheere-Terpstra test (standard *Z* value) and the chi-squared test with linear-by-linear association (*x* ^2^ value) were used, as appropriate.

Second, Pearson’s correlation was applied to assess univariate correlation between serum ADA levels and nerve conduction indices. Moreover, given that HbA1c may exert an effect on these correlations, partial correlation was used to adjust the effect of HbA1c.

Third, to explore the independent effects of serum ADA levels on nerve conduction indices, we applied multivariable linear regression analyses to control for other clinical covariates. Meanwhile, to explore the independent effects of serum ADA levels on the risk of DPN, we applied multivariable logistic regression analyses to determine unadjusted and adjusted odds ratios (ORs) and 95% confidence intervals (CIs) for DPN when each 5 U/L was increased in serum ADA levels.

Finally, we applied receiver operating characteristic (ROC) curves to assess the diagnostic capability of serum ADA levels to confirm DPN and to calculate the optimal cut-off value of serum ADA levels to discriminate DPN. With this optimal cut-off value of serum ADA levels, sensitivity, specificity, positive predictive value (PPV), negative predictive value (NPV), positive likelihood ratio (PLR) and negative likelihood ratio (NLR) were also provided. We also used ROC analysis to compare the capability of serum ADA and HbA1c to discriminate DPN by the methods of DeLong et al.

We used SPSS for Windows (Version 25.0, IBM Corp.) to pool and analyse clinical data, and statistical significance was identified when the *p* value<0.05.

## Results

### Clinical features of recruited patients

**Figure 1:**
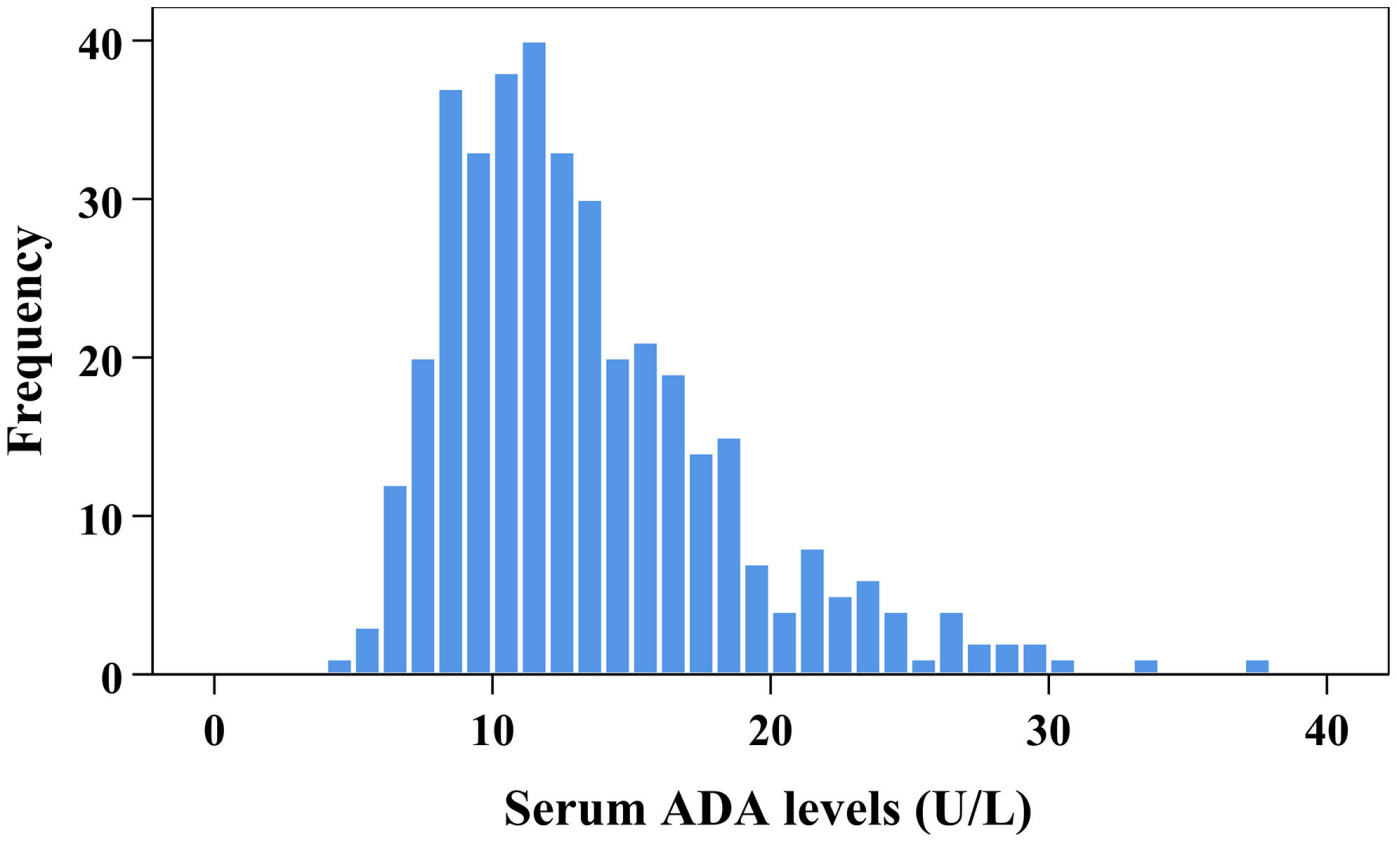
Frequency distribution of serum ADA levels.

The frequency distribution of serum ADA levels is displayed **Figure 1**. The clinical features of all eligible patients are shown in **Table 1**. Age, SBP, diabetes duration, current smoking, ALT, GGT, CysC and HbA1c increased with ascending serum ADA quartile, whereas eGFR and insulin sensitivity index (IS-CP) decreased. Conversely, the female distribution, BMI, DBP, hypertension history, statin use, albumin, TBI, lipid profiles and UA showed no differences among the four subgroups. Regarding antidiabetic treatments, insulin use tended to be increased with ascending serum ADA quartile. However, drug naiveness and the use of secretagogues, metformin, TZDs, AGIs, DPP-4Is, SGLT-2Is and GLP-1RAs were comparable among the four subgroups. With increasing quartiles of serum ADA levels, the composite *Z* score of latency increased, whereas that of amplitude and NCV decreased significantly. After DPN assessment, 24.5% (n=94) of the recruited patients were determined to have DPN. The prevalence of DPN in Q1, Q2, Q3 and Q4 of serum ADA levels was 15.3%, 16.0%, 22.9% and 43.8%, respectively (*p for trend*<0.001).

**Table 1:** Clinical features of the recruited patients.

Variables	Total	Quartiles of serum ADA levels				*Test statistic*	*p for trend*
		Q1	Q2	Q3	Q4		
Serum ADA (U/L) (range)	13.26 ± 5.22 (4.0–37.0)	7.95 ± 1.13 (4.0–9.5)	10.83 ± 0.74 (9.6–12.1)	13.81 ± 1.06 (12.2–15.9)	20.51 ± 4.30 (16.0–37.0)	–	–
*N*	384	98	94	96	96	–	–
Age (year)	51.7 ± 11.3	48.4 ± 10.3	51.1 ± 10.9	53.4 ± 11.7	54.0 ± 11.7	14.071**^a^**	<0.001
Female, n (%)	154 (40.1)	34 (34.7)	41 (43.6)	38 (39.6)	41 (42.7)	0.824	0.364
BMI (kg/m^2^)	25.08 ± 3.24	24.69 ± 2.92	25.65 ± 3.50	24.90 ± 2.99	25.11 ± 3.49	0.141**^a^**	0.708
SBP (mmHg)	133.3 ± 16.6	128.0 ± 17.3	134.8 ± 16.4	133.4 ± 16.2	137.2 ± 15.1	12.721**^a^**	<0.001
DBP (mmHg)	79.6 ± 10.5	78.9 ± 10.6	80.9 ± 9.3	79.7 ± 11.1	79.1 ± 10.8	0.016**^a^**	0.899
Diabetes duration (year)	5.0 (1.0–10.0)	3.0 (1.0–7.0)	5.5 (1.0–10.0)	7.5 (1.0–10.0)	7.0 (1.0–12.0)	2.831**^b^**	0.005
Antidiabetic treatments							
Drug naive, *n* (%)	55 (14.3)	13 (13.3)	11 (11.7)	16 (16.7)	16 (15.6)	0.562**^c^**	0.453
Insulin, *n* (%)	160 (41.7)	31 (31.6)	30 (31.9)	50 (52.1)	49 (51.0)	12.136**^c^**	<0.001
Secretagogues, *n* (%)	169 (44.0)	36 (36.7)	45 (47.9)	47 (49.0)	41 (42.7)	0.736**^c^**	0.391
Metformin, *n* (%)	189 (49.2)	50 (51.0)	46 (48.9)	54 (56.3)	39 (40.6)	1.093**^c^**	0.296
TZDs, *n* (%)	70 (18.2)	17 (17.3)	25 (26.6)	13 (13.5)	15 (15.6)	1.031**^c^**	0.310
AGIs, *n* (%)	54 (14.1)	13 (13.3)	9 (9.6)	14 (14.6)	18 (18.8)	1.803**^c^**	0.179
DPP-4Is, *n* (%)	56 (14.6)	17 (17.3)	12 (12.8)	14 (14.6)	13 (13.5)	0.367**^c^**	0.545
SGLT-2Is, *n* (%)	18 (4.7)	7 (7.1)	5 (5.3)	5 (5.2)	1 (1.0)	3.659**^c^**	0.056
GLP-1RAs, *n* (%)	24 (6.1)	6 (6.1)	3 (3.2)	4 (4.2)	11 (11.5)	2.322**^c^**	0.128
Hypertension, *n* (%)	151 (39.3)	34 (34.7)	43 (45.7)	35 (36.5)	39 (40.6)	0.159**^c^**	0.690
Current smoking, *n* (%)	115 (29.9)	23 (23.5)	26 (27.7)	28 (29.2)	38 (39.6)	5.705**^c^**	0.017
Statins uses, *n* (%)	116 (30.2)	27 (27.6)	27 (28.7)	34 (35.4)	28 (29.2)	0.307**^c^**	0.580
ALT (U/L)	19 (13–29)	18 (13–25)	16 (11–24)	21 (13–30)	21 (14–33)	2.494**^b^**	0.013
AST (U/L)	16 (14–22)	16 (13–19)	15 (13–20)	17 (14–23)	18 (15–26)	4.503**^b^**	<0.001
GGT (U/L)	29 (20–47)	27 (19–40)	27 (20–42)	31 (19–52)	33 (22–54)	2.984**^b^**	0.003
TBI (μmol/L)	10.5 (7.7–13.6)	10.2 (7.6–12.9)	10.0 (7.3–12.7)	10.9 (7.8–13.2)	11.8 (7.9–15.4)	1.838**^b^**	0.066
Albumin (g/L)	38.9 ± 3.8	39.1 ± 3.5	38.7 ± 3.7	39.0 ± 3.8	38.6 ± 3.8	0.439**^a^**	0.508
TG (mmol/L)	1.66 (1.05–2.51)	1.55 (1.02–2.41)	1.47 (0.99–2.30)	1.79 (1.13–2.84)	1.69 (1.06–2.75)	1.251**^b^**	0.211
TC (mmol/L)	4.41 ± 0.96	4.41 ± 0.83	4.42 ± 0.87	4.55 ± 1.09	4.28 ± 1.02	0.288**^a^**	0.592
HDLC (mmol/L)	1.18 ± 0.36	1.16 ± 0.26	1.19 ± 0.25	1.17 ± 0.32	1.18 ± 0.54	0.094**^a^**	0.759
LDLC (mmol/L)	2.72 ± 0.86	2.76 ± 0.75	2.76 ± 0.72	2.73 ± 1.00	2.69 ± 0.91	0.454**^a^**	0.501
UA (μmol/L)	302 ± 92	299 ± 76	300 ± 93	305 ± 100	303 ± 100	0.153**^a^**	0.696
CysC (mg/L)	0.82 (0.70–1.00)	0.75 (0.59–0.90)	0.80 (0.70–1.00)	0.84 (0.70–1.00)	1.00 (0.88–1.10)	6.818**^b^**	<0.001
eGFR (mL/min/1.73m^2^)	119.5 ± 30.7	126.2 ± 31.0	117.7 ± 29.2	119.1 ± 29.8	115.0 ± 31.9	5.344**^a^**	0.021
IS-CP	2.14 (1.39–3.32)	2.29 (1.63–3.60)	2.11 (1.51–3.25)	2.10 (1.30–3.61)	1.92 (1.07–3.26)	–2.468**^b^**	0.014
HbA1c (%)	8.08 ± 1.16	7.81 ± 1.24	8.03 ± 1.23	7.99 ± 1.00	8.50 ± 1.06	15.503**^a^**	<0.001
Composite *Z* score of latency	0.025 ± 0.602	–0.152 ± 0.559	–0.060 ± 0.589	0.039 ± 0.563	0.274 ± 0.618	27.008**^a^**	<0.001
Composite *Z* score of amplitude	–0.024 ± 0.643	0.106 ± 0.617	0.017 ± 0.576	–0.018 ± 0.677	–0.201 ± 0.670	10.904**^a^**	<0.001
Composite *Z* score of NCV	–0.022 ± 0.655	0.165 ± 0.589	0.063 ± 0.590	–0.036 ± 0.683	–0.282 ± 0.677	24.647**^a^**	<0.001
DPN, *n* (%)	94 (24.5)	15 (15.3)	15 (16.0)	22 (22.9)	42 (43.8)	22.064**^c^**	<0.001

**^a^** Linear polynomial contrasts of ANOVA (F value), **^b^** Jonckheere-Terpstra test (Z value) or **^c^** linear-by-linear association of chi-squared test (x^2^ value) were performed as appropriate.

### Correlations of serum ADA with nerve conduction indices

**Figure 2:**
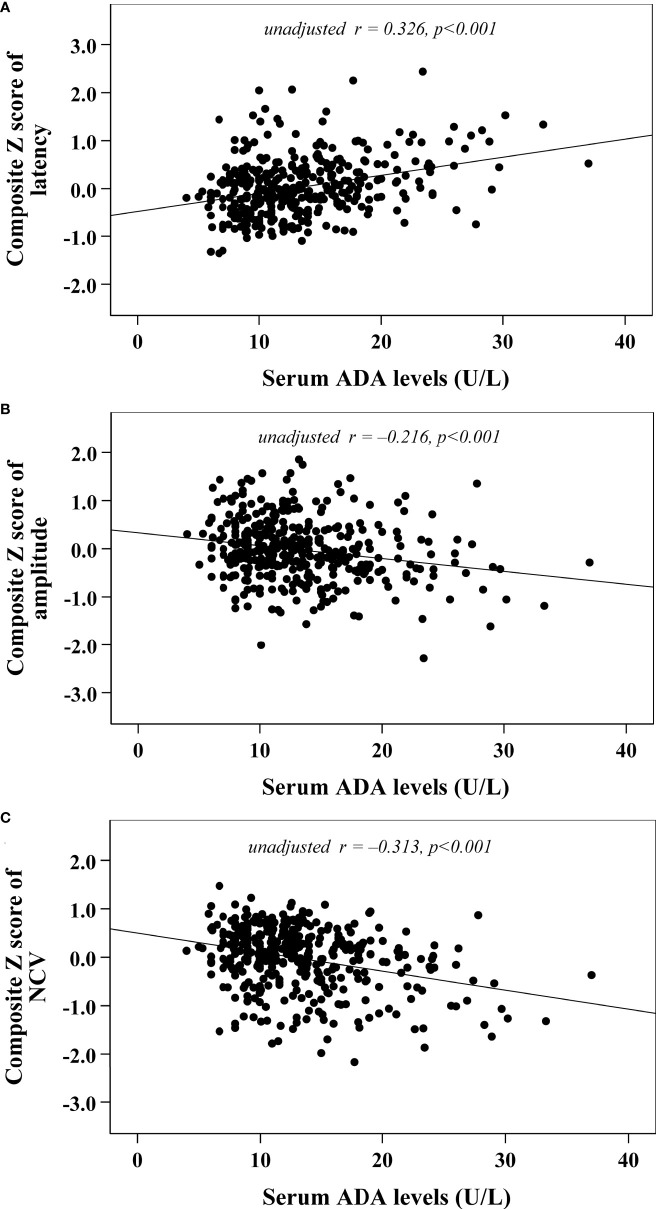
Correlations between serum ADA levels and nerve conduction indices **(A)** composite Z score of latency; **(B)** composite Z score of amplitude; **(C)** composite Z score of NCV).

**Figure 3:**
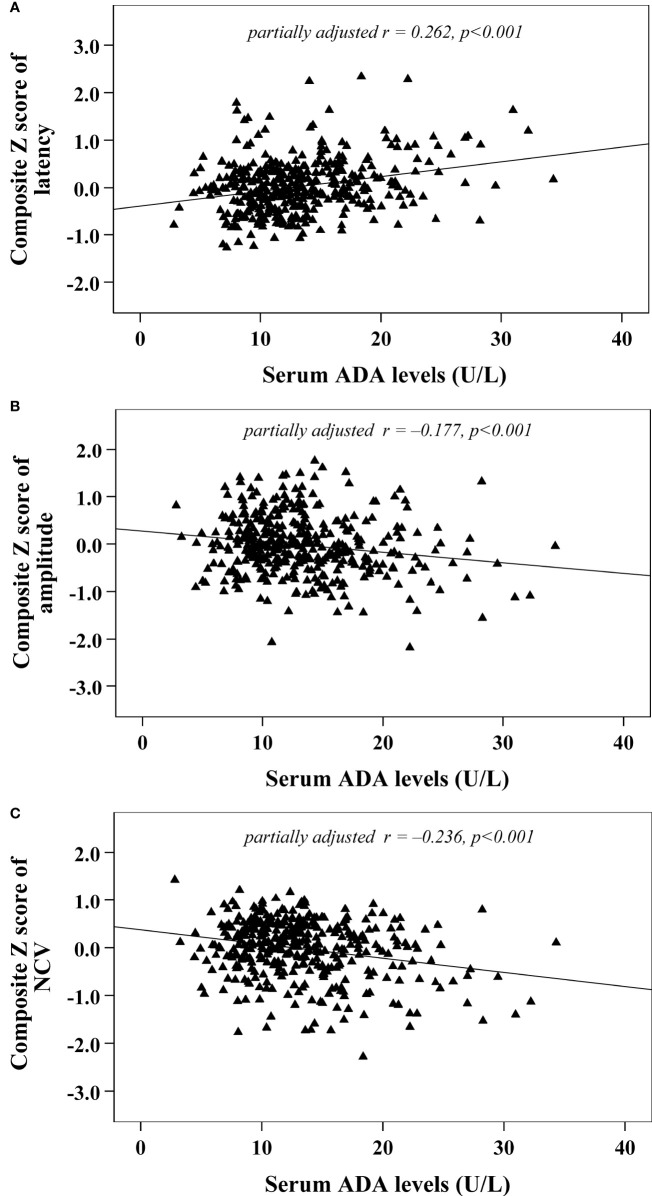
Correlations between serum ADA levels and nerve conduction indices after adjusting for HbA1c **(A)** composite Z score of latency; **(B)** composite Z score of amplitude; **(C)** composite Z score of NCV).

Univariate correlation analysis demonstrated that serum ADA levels were correlated with composite *Z* scores of nerve latency, amplitude and NCV (*r*= 0.326, –0.216 and –0.313, respectively, *p*<0.001) (**Figure 2**). After controlling for the potential effect of HbA1c on these correlations by partial correlation analysis, serum ADA levels remained linked to composite *Z* scores of nerve latency, amplitude and NCV (*r*= 0.262, –0.177 and –0.236, respectively, *p*<0.001) (**Figure 3**).

Moreover, we used multivariable linear regression analyses to determine the effects of serum ADA levels on nerve conduction indices (**Table 2**). After gradually adjusting for other clinical covariates (from model 0 to model 3), serum ADA levels remained independently associated with nerve conduction indices. The fully adjusted model 3 demonstrated that serum ADA levels were independently and positively associated with composite *Z* score of latency (*β*=0.263, *t*=5.273, *p*<0.001) and independently and negatively associated with composite *Z* score of amplitude (*β*= –0.126, *t*= –2.352, *p*=0.019) and NCV (*β*= –0.201, *t*= –3.841, *p*<0.001), respectively.

**Table 2:** Impacts of serum ADA levels on nerve conduction indices by multivariable linear regression analysis.

Models	B (95% CI)	*β*	*t*	*p*	Adjusted *R^2^*
**Composite Z score of latency**					
Model 0:	0.038 (0.027 to 0.049)	0.326	6.747	<0.001	0.106
Model 1	0.033 (0.024 to 0.044)	0.286	6.298	<0.001	0.295
Model 2	0.030 (0.019 to 0.040)	0.260	5.474	<0.001	0.436
Model 3	0.030 (0.019 to 0.041)	0.263	5.273	<0.001	0.439
**Composite Z score of amplitude**					
Model 0:	–0.027 (–0.039 to –0.015)	–0.216	–4.331	<0.001	0.047
Model 1	–0.019 (–0.030 to –0.007)	–0.152	–3.179	0.002	0.216
Model 2	–0.016 (–0.028 to –0.003)	–0.127	–2.433	0.015	0.320
Model 3	–0.016 (–0.029 to –0.003)	–0.126	–2.352	0.019	0.355
**Composite Z score of NCV**					
Model 0:	–0.039 (–0.051 to –0.027)	–0.313	–6.432	<0.001	0.098
Model 1	–0.034 (–0.046 to –0.022)	–0.268	–5.483	<0.001	0.181
Model 2	–0.026 (–0.038 to –0.014)	–0.207	–4.093	<0.001	0.363
Model 3	–0.025 (–0.038 to –0.012)	–0.201	–3.841	<0.001	0.384

Model 0: unadjusted.

Model 1: adjusted for age, sex, diabetes duration, BMI, SBP, DBP, hypertension, current smoking and statin treatment.

Model 2: additionally adjusted for ALT, AST, GGT, TBI, albumin, lipid profiles, UA, eGFR, IS-CP and HbA1c.

Model 3: additionally adjusted for antidiabetic treatments.

### Risks for DPN with increasing serum ADA

We applied multivariable logistic regression analyses to determine the independent effects of serum ADA levels on the risk of DPN (**Table 3**). In the unadjusted Model 0, each 5 U/L increase in serum ADA levels may account for a 2.027-fold increased OR of having DPN (95% CI: 1.602–2.565, *p*<0.001, Nagelkerke R^2^ = 0.145). After gradually adjusting for other clinical covariates (from model 0 to model 3), increased serum ADA levels remained independently associated with increased risks for DPN. The fully adjusted model 3 demonstrated that each 5 U/L increase in serum ADA levels may account for a 1.781-fold increased adjusted OR of having DPN (95% CI: 1.271–2.495, *p*<0.001, Nagelkerke R^2^ = 0.416).

**Table 3:** Impacts of serum ADA levels (per 5 U/L increase) on the risk of DPN by multivariable logistic regression analysis.

Models	OR (95% CI)	*p*	Nagelkerke R^2^
Model 0:	2.027 (1.602–2.565)	<0.001	0.145
Model 1	1.976 (1.537–2.540)	<0.001	0.200
Model 2	1.867 (1.362–2.561)	<0.001	0.399
Model 3	1.781 (1.271–2.495)	0.001	0.416

Model 0: unadjusted.

Model 1: adjusted for age, sex, diabetes duration, BMI, SBP, DBP, hypertension, current smoking and statin treatment.

Model 2: additionally adjusted for ALT, AST, GGT, TBI, albumin, lipid profiles, UA, eGFR, IS-CP and HbA1c.

Model 3: additionally adjusted for antidiabetic treatments.

### Potential capability of serum ADA to discriminate DPN

**Figure 4:**
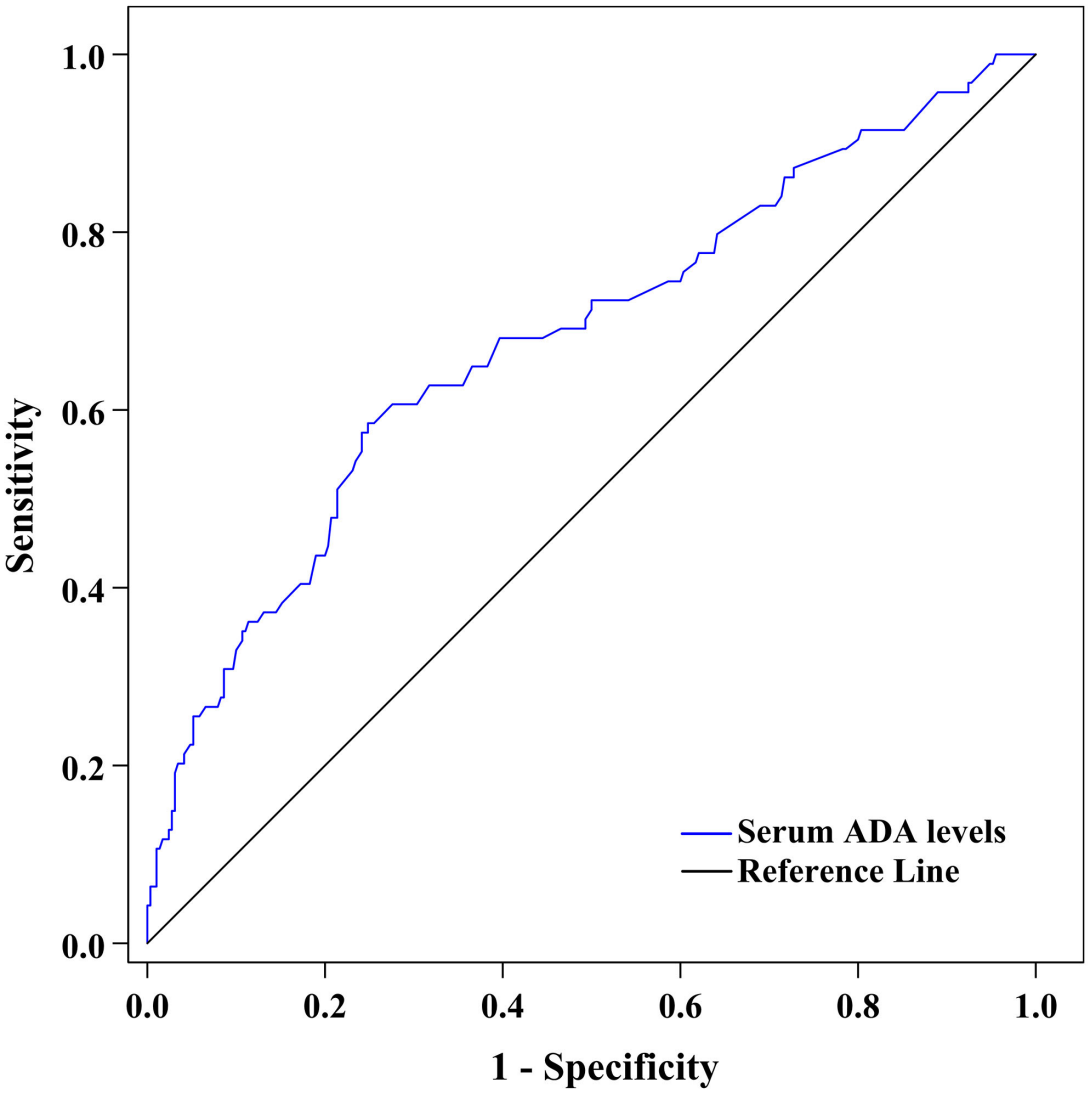
ROC curve exhibiting the capability of serum ADA levels to discriminate DPN (AUC was 0.685 [95% CI: 0.636–0.731], optimal cut-off value was ≥14.2 U/L, Youden index was 0.351, sensitivity was 59.57%, and specificity was 75.52%).

**Figure 4** shows the ability of serum ADA to discriminate DPN after ROC curve analysis. The area under the ROC curve (AUC) of serum ADA was 0.685 (95% CI: 0.636–0.731). Additionally, ROC analysis determined that the optimal cut-off value of serum ADA levels to discriminate DPN was ≥14.2 U/L, with a sensitivity of 59.57% (95% CI: 48.95%–69.58%), a specificity of 75.52% (95% CI: 70.15%–80.36%), a Youden index of 0.351, a PPV of 44.09% (95% CI: 37.77%–50.61%), an NPV of 85.21% (95% CI: 81.72%–88.14%), a PLR of 2.433 (95% CI: 1.873–3.162) and an NLR of 0.535 (95% CI: 0.415–0.690).

### Comparison of serum ADA and HbA1c to discriminate DPN

**Figure 5:**
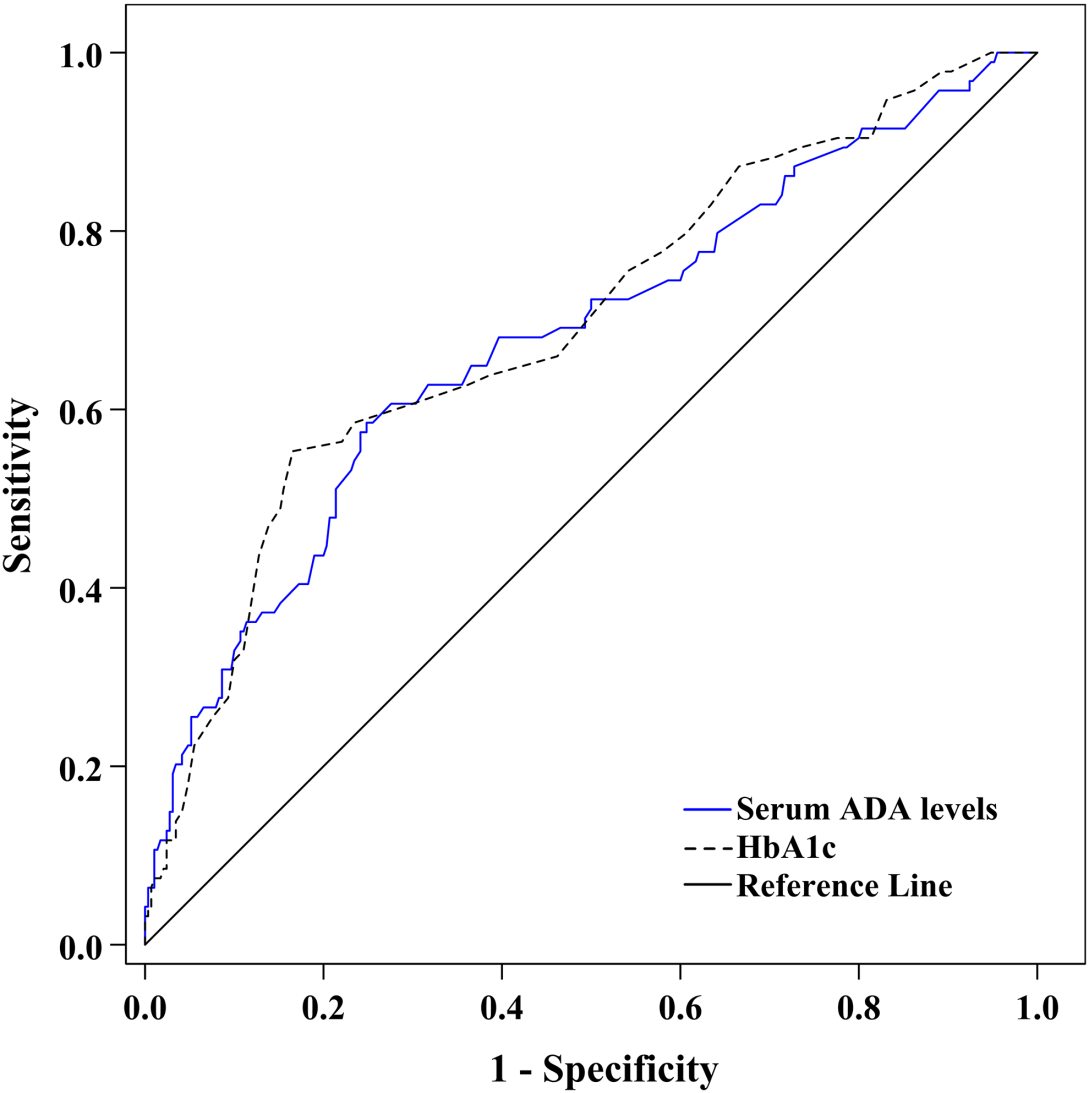
ROC curve comparing the capability of serum ADA levels with HbA1c to discriminate DPN.

Because HbA1c was identified as a traditional risk factor for DPN in our studies (22, 23), we used ROC analysis to compare the capability of serum ADA and HbA1c to discriminate DPN (**Figure 5**). The AUC of HbA1c in our present study was 0.699 (95% CI: 0.650–0.745). After comparison with HbA1c, the capability of serum ADA to discriminate DPN was comparable to that of HbA1c (AUC difference of 0.014 [95% CI: –0.075 to 0.103], *Z*=0.310, *p*= 0.757).

## Discussion

In the present study, we characterized the cross-sectional association of serum ADA levels with nerve conduction parameters and risk of DPN in 384 patients with T2D. The primary contributions of the study are described as follows: first, increased serum ADA levels were independently associated with higher nerve action potential onset latency and lower action potential amplitude and NCV; second, each 5 U/L increase in serum ADA levels may account for a 1.78-fold increased risk of having DPN; third, serum ADA levels ≥14.2 U/L was the optimal cut-off value to discriminate DPN, with a sensitivity and specificity of 59.57% and 75.52%, respectively; fourth, compared to HbA1c, a well-established risk factor for DPN, we found that serum ADA levels and HbA1c were comparable in their ability to discriminate DPN.

ADA, ubiquitously expressed throughout the body, has enzymatic and extraenzymatic properties to maintain metabolic homeostasis and immune balance (28). Increased levels of ADA expression have been demonstrated to be involved in immunoinflammatory and metabolic diseases (28). Previous basic and clinical studies have shown that increased serum ADA activity assumes an important role in the pathophysiological processes of organ-specific and systemic autoimmune diseases (29, 30); the former includes myasthenia gravis and Graves’ disease, and the latter includes rheumatoid arthritis and systemic lupus erythematosus. In addition, increased serum ADA levels were shown to have potential value in the diagnosis and surveillance of these autoimmune diseases (29). Moreover, serum ADA levels were also found to be elevated in inflammatory diseases, such as ectopic pregnancy, gestational diabetes mellitus, preeclampsia, and inflammatory bowel disease (31–34). In addition, patients with increased cardiometabolic risks are always characterized by elevated serum ADA levels, and these cardiometabolic risks include higher BMI (35), increased TC (36), hypertension (16), atherosclerosis (13), thrombosis (14), coronary artery calcification (37) and T2D (38). Furthermore, in our previous studies and other previous studies, elevated serum ADA levels are not only involved in impaired pancreatic β-cell function in T2D (39) but also contribute to several diabetic complications, such as diabetic retinopathy (40), diabetic nephropathy (41), and prolonged heart QT interval in T2D (42). In our present study, serum ADA levels were observed to be positively correlated with ageing, higher SBP, longer diabetes duration, and increased levels of ALT, AST, GGT, CysC and HbA1c and negatively correlated with eGFR and insulin sensitivity index (IS-CP). Most importantly, our present study revealed that increased serum ADA levels may be a potential risk factor for DPN in patients with T2D, independent of traditional cardiometabolic risk factors.

In previous studies, increased serum ADA levels were associated with higher BMI (35) and increased TC (36). However, in our present study, lipid metabolism measures and BMI were not correlated with serum ADA levels. The recruited patients with T2D received antidiabetic treatments, such as metformin, sodium-glucose cotransporter-2 inhibitors (SGLT-2Is), and glucagon-like peptide-1 receptor agonists (GLP-1RAs), which may have roles in decreasing BMI. Meanwhile, 30.2% of recruited patients also received statin treatments. These interventions may attenuate or eliminate the association of lipid metabolism measures and BMI with serum ADA levels.

Although DPN is initiated and progresses in the context of diabetes, the pathophysiological process of DPN involves cumulative effects and interactions of multiple cardiometabolic risk factors, especially in patients with T2D (20). Exposure to deranged glycemic control remains a fundamental risk factor for DPN (20). In our previous studies and other previous studies, glycemic disorders were demonstrated to contribute to DPN in patients with T2D, including daily glycemic fluctuation (increased mean amplitude of glycemic excursions) (43), weekly glycemic fluctuation (decreased plasma 1,5-anhydro-d-glucitol) (25), long-term hyperglycemia (increased HbA1c) (44), long-term glycemic fluctuation (increased HbA1c variability) (45) and overall glycemic exposure (decreased time in glucose range, TIR) (46). In addition to deranged glycemic control, the development of DPN is also inseparable from inflammatory environments caused by the complex interaction of multiple cardiometabolic risk factors, such as smoking, obesity, insulin resistance, hypertension, and dyslipidemia (47). Several transversal and longitudinal studies have shown that increased chronic inflammatory indicators, including serum C-reactive protein (hs-CRP) (48), tumor necrosis factor-α (TNF-α), interleukin-6 (IL-6), soluble intercellular adhesion molecule (sICAM-1) (49), circulating neutrophil-to-lymphocyte ratio (50) and total leukocyte count (51), are related to a higher risk of DPN. Normal serum ADA activity is critical for the maintenance of metabolic and immune homeostasis. In the present study, we found that increased serum ADA levels were associated with impaired nerve conduction indices and an increased risk of DPN in patients with T2D. We also calculated that levels of serum ADA ≥14.2 U/L had a sensitivity of 59.57% and a specificity of 75.52% to discriminate DPN. Therefore, increased serum ADA levels may be an emerging risk factor for DPN in T2D.

There are some possible mechanisms proposed to interpret the relationship between increased serum ADA levels and DPN in T2D. T2D is a chronic inflammatory disease (52), and the inflammatory microenvironment paves the way for the development of DPN (20). Increased levels of ADA expression have been shown to participate in immunoinflammatory and metabolic disturbances (12), which in turn favor the formation of the inflammatory microenvironment. On the one hand, ADA represents an essential checkpoint *via* its enzymatic property to terminate adenosine levels (28). Extracellular adenosine levels regulate vascular homeostasis by maintaining platelet function and the proper order of inflammation, smooth muscle and endothelial cells (53). Hence, increased serum ADA levels could reflect the severity of endothelial activation and vascular inflammation by downregulation of adenosine signaling (12). On the other hand, ADA also plays an extraenzymatic role in the regulation of cell-to-cell interactions (54). ADA is a costimulatory molecule that bridges cells expressing dipeptidyl peptidase IV (CD26) and ADA-anchoring proteins and forms trimeric complexes (55). Increased activity of ADA can induce hyperpermeability of the endothelium and increase costimulatory interactions between endothelial and immune cells. These costimulatory responses subsequently lead to an increased number of T helper (Th) cells and an increased expression of proinflammatory adipokines of Th cells, such as TNF-α and IL-6 (56). In addition, increased activity of ADA can also promote the differentiation of monocytes into macrophages through the assistance of activated Th cells (57). Inflammatory injury occurs in neuronal, glial and microvascular endothelial cells and stimulates macrophage activation, all of which can result in impairment of nerve function and neuropathy (20, 47). Moreover, ADA is a regulator of insulin action on glycemic metabolism (19). Increased activity of ADA can directly contribute to insulin resistance, which in turn impairs neurotrophic signaling and mitochondrial dysfunction, ultimately leading to nerve dysfunction and neuropathy (47, 58).

The present study should be addressed in light of a few limitations. First, due to the cross-sectional design, our present data are not sufficient to establish a causal relationship between increased serum ADA levels and DPN. In this regard, a longitudinal study is currently being conducted to compensate for this deficiency. In future follow-up study, we will address the following questions (1): Can higher serum ADA levels at baseline predict the incidence of DPN over time in a subgroup without DPN? (2) Are changes in serum ADA levels associated with changes in nerve conduction indices over time in these patients? Second, our study was restricted to the Chinese population with T2D in a single center, and the associations of increased serum ADA levels with nerve conduction indices and risk of DPN were relatively weak. Although this is a large cohort study, the findings may have limited generalizability. Third, we did not analyze the relationship between serum ADA levels and the severity of DPN evaluated by the scoring of DPN signs/symptoms in our present study, such as the Michigan Neuropathy Screening Instrument (MNSI) and the modified Toronto Clinical Neuropathy Scale (mTCNS). Fourth, we did not use a quantitative sensory test (QST) or intraepidermal nerve fiber density testing in skin biopsy to evaluate small fiber neuropathy. Fifth, the levels of serum ADA may correlate with markers of systemic inflammation, but we did not assess the effect of inflammatory markers on the relationship between serum ADA and DPN. Sixth, in the human body, ADA has two isoenzymes, i.e., ADA1 and ADA2. We did not detect the ADA1 and ADA2 isoenzymes, and we cannot distinguish the possible differences in the role of isoenzymes in DPN.

## Conclusion

In summary, increased serum ADA levels may be a potential risk factor for DPN in patients with T2D.

## Data availability statement

The original contributions presented in the study are included in the article/supplementary material. Further inquiries can be directed to the corresponding author.

## Ethics statement

The studies involving human participants were reviewed and approved by First People’s Hospital of Nantong. The patients/participants provided their written informed consent to participate in this study.

## Author contributions

J-BS and X-QW initiated and acquired funding for the series. CY and J-BS designed this study. D-MZ coordinated and supervised the study. CY, LZ, FX, L-HZ, X-HW, C-HW, L-YN and X-LZ recruited patients and collected the data. CY and J-BS analysed the data and interpreted the results. CY drafted the manuscript. All authors contributed to the article and approved the submitted version.

## Funding

The study was funded by Social Development Projects of Nantong (MS22015065, MS12019019, HS2020005) and Medical Research Project of Jiangsu Health Commission (QNRC2016408).

## Conflict of interest

The authors declare that the research was conducted in the absence of any commercial or financial relationships that could be construed as a potential conflict of interest.

## Publisher’s note

All claims expressed in this article are solely those of the authors and do not necessarily represent those of their affiliated organizations, or those of the publisher, the editors and the reviewers. Any product that may be evaluated in this article, or claim that may be made by its manufacturer, is not guaranteed or endorsed by the publisher.

## Abbreviations

T2D, type 2 diabetes; DPN, diabetic peripheral neuropathy; ADA, adenosine deaminase; SBP/DBP, systolic/diastolic blood pressure; BMI, body mass index; TZDs, thiozolindiones; AGIs, α-glucosidase inhibitors; DPP-4Is, dipeptidyl peptidase-4 inhibitors; SGLT-2Is, sodium-glucose cotransporter-2 inhibitors; GLP-1RAs, glucagon-like peptide-1 receptor agonists; ALT, alanine aminotransferase; AST, aspartate aminotransferase; GGT, gamma-glutamyl transpeptidase; TBI, total bilirubin; TG, triglycerides; TC, total cholesterol; HDLC, high-density lipoprotein cholesterol; LDLC, low-density lipoprotein cholesterol; UA, uric acid; CysC, cystatin C; eGFR, estimated glomerular filtration rate; HbA1c, glycosylated hemoglobin A1c; IS-CP, insulin sensitivity calculated based on fasting C-peptide; MN, median nerve; UN, ulnar nerve; CPN, common peroneal nerve; PTN, posterior tibial nerve; SN, sural nerve; SPN, superficial peroneal nerve; NCV, nerve conduction velocity; PPV, positive predictive value; NPV, negative predictive value; PLR, positive likelihood ratio; NLR, negative likelihood ratio.
